# Notes on Preparations for the Sick

**Published:** 1901-09

**Authors:** 


### Notes on Preparations for the Sick.

**Izal Cream; Izal Medical Soap; Izal Oil Perles** (2 minims in each, in oil).—Newton, Chambers & Co., Thorncliffe, Sheffield. —The various Izal preparations are now so well known as to need no commendation. The above samples have been forwarded to us.

The Cream contains $2\frac{1}{2}$ per cent. of Izal, and is a very convenient antiseptic application for many surgical purposes.

The Soap contains 10 per cent; it is superfatted, soft and agreeable, and is useful for disinfecting the hands before and after operations, and for general antiseptic purposes in the sick-room.

The Perles are for internal use, where the action of intestinal antiseptics is desired. They should be useful in cases requiring the internal administration of an easily borne non-irritating antiseptic.

The researches of Klein, Delépine, Bruce Clarke, Tunnicliffe, and others, shew that medical Izal, although non-poisonous and non-irritant, is four times stronger than absolute phenol; and when used in a strength of 1 in 200 it will destroy every variety of the most resistant bacilli in five minutes.

---

**Tabloids.**—Burroughs, Wellcome & Co., London.—The following new tabloids have been forwarded to us:—

Ergotin and Strychnine,
Galbanum compound,
Apomorphine hydrochloride and Strychnine hydrochloride,
Hyoscine compound A, and Hyoscine compound B,
Sodium cacodylate.

The two former are sugar-coated; the latter are intended for hypodermic use.

The Ergotin and Strychnine compound contains three grains of ergotin and one-thirtieth of a grain of strychnine sulphate. Where these drugs are indicated, the tabloid is a convenient form ensuring accuracy of dosage and efficiency in therapeutic result.

The Galbanum compound contains four grains of the Pharmacopeal preparation known as the "compound pill of Galbanum," which has long been recognised as an efficient carminative and anti-spasmodic. The pill is perhaps a little out-of-date and apt to be forgotten, but possibly the presentation of the drug in tabloid form may revive its old reputation.

The Apomorphine and strychnine hydrochloride hypodermic tabloid contains gr. $\frac{1}{10}$ of the former and gr. $\frac{1}{60}$ of the latter. Where the emetic property of apomorphine is desired, one of these tabloids should be sufficient, if given hypodermically; but they may be administered much more freely by the mouth in cases where an expectorant is desirable. The combination given by the mouth should be very useful if taken every two or three hours to encourage expectoration in cases of bronchitis.

The Hyoscine compound A has the following composition:—

| | |
|---|---|
| Hyoscine hydrobromide ... | gr. $\frac{1}{100}$ |
| Morphine sulphate ... ... | gr. $\frac{1}{6}$ |
| Atropine sulphate ... ... | gr. $\frac{1}{180}$ |

The compound B has :—

| | | |
|---|---|---|
| Hyoscine hydrobromide | ... | gr. $\frac{1}{100}$ |
| Morphine sulphate | ... ... | gr. $\frac{1}{4}$ |
| Atropine sulphate | ... ... | gr. $\frac{1}{150}$ |

Hyoscine is well known to be a somewhat uncertain drug, which must be given with caution, and of which the dose must be regulated with care. Its efficacy is likely to be increased by the addition of small doses of morphia and atropine, and the combination is giving good results.

The Sodium cacodylate is in the form of tabloid (gr. $\frac{1}{4}$) of the ordinary compressed type; and also in tubes for hypodermic use. Experience has shown that very large doses of arsenic may safely be given in this form; but the therapeutic efficacy of this compound is still *sub-judice*, and these tabloids are likely to assist in the solution of the question.

